# 3D Muography for the Search of Hidden Cavities

**DOI:** 10.1038/s41598-019-39682-5

**Published:** 2019-02-27

**Authors:** Luigi Cimmino, Guglielmo Baccani, Pasquale Noli, Lucio Amato, Fabio Ambrosino, Lorenzo Bonechi, Massimo Bongi, Vitaliano Ciulli, Raffaello D’Alessandro, Mariaelena D’Errico, Sandro Gonzi, Barbara Melon, Gianluca Minin, Giulio Saracino, Luca Scognamiglio, Paolo Strolin, Lorenzo Viliani

**Affiliations:** 1 University of Naples Federico II., Naples, Italy; 2 INFN sezione di Napoli., Naples, Italy; 3 University of Florence, Florence, Italy; 4 INFN sezione di Firenze, Florence, Italy; 5 TECNO-IN S.P.A., Naples, Italy; 6 Associazione Culturale Borbonica Sotterranea, Naples, Italy

**Keywords:** Experimental particle physics, Imaging techniques

## Abstract

Muography (or muon radiography) is a technique that exploits the penetration capability of muons, elementary particles similar to electrons but with a mass about 200 times larger. High energy muons are naturally produced in the interactions of cosmic rays with the Earth atmosphere. The measurement of their absorption in matter allows the imaging of the inner structure of large bodies. The technological developments in the detection of elementary particles have opened the way to its application in various fields, such as archaeology, studies of geological structures, civil engineering and security issues. We have developed a new approach to the three-dimensional muography of underground structures, capable of directly localising hidden cavities and of reconstructing their shape in space. Our measurements at Mt. Echia, the site of the earliest settlement of the city of Naples in the 8th century BC, have led us to the discovery of a hidden underground cavity, whose existence was not evident with the usual two-dimensional muography graphs. We demonstrate here that our original approach definitely enhances muography discovery potential, especially in case of complex underground systems.

## Introduction

In all fields, imaging techniques capable of seeing inside physical bodies have brought an extraordinary progress to the power of investigation procedures. Muography is an imaging technique that profits from the penetrating power of elementary particles called muons, similar to electrons but with a mass about two hundred times larger. Muons are naturally produced in the interactions of cosmic rays with the Earth atmosphere. A conspicuous flux of muons is constantly hitting the Earth surface from all directions, from the Zenith to almost horizontal, providing an abundant “light” source for muography.

In absorption muography, a muon tracker is located downstream of the body under investigation. By tracking back the muon trajectories, one obtains an angular map of their flux as seen from the location of the muon tracker itself. A comparison with the muon flux impinging on the Earth surface provides a map of the muon transmission (or equivalently absorption) in the traversal of the body being investigated. As the penetration of muons in matter depends on its density, such a map can provide a muographic image of its internal structures. These could be cavities or high-density zones. Muography (or muon radiography) is thus in principle similar to X-ray radiography, but capable of probing the interior of large bodies, thanks to the penetrating power of muons which is much higher than that of X-rays.

The first muography was performed by the Nobelist Louis Alvarez in 1970, who disproved the possible existence of a hidden burial chamber in Chefren’s Pyramid^1^. Almost forty years later, muography was applied to the investigation of the inner structures of volcanic edifices^2–13^, facing the challenge posed by the observation of the low muon fluxes surviving the traversal of a large rock thickness typical of this application. However, the problem of low fluxes does not affect a very wide range of applications (from archaeology to risk assessment and prevention in civil engineering or geology), where the thickness of matter being traversed is at most several tens of meters^14–22^. The results obtained by the ScanPyramids project at the Khufu’s Pyramid^23^ and by our group in exploring underground cavities at Mt. Echia in ancient Naples^24^ belong to this domain.

Mt. Echia (also called Pizzofalcone) is the site of the earliest settlement of the city of Naples in 8th century BC. It is a headland with a maximum altitude of about 60 m a.s.l. and mainly consists of yellow tuff, a soft volcanic rock. In the course of history a very complex system of underground tunnels and cavities has been excavated and used for an astonishing variety of purposes, thus carrying a wealth of peculiar information on the past life of the city. This system of cavities has recently become the subject of systematic investigations and is partially available to visitors who are curious about the underground history of Naples, with access through the so-called Bourbon Tunnel that was excavated around the middle of the 19th century.

In our study of underground cavities at Mt. Echia^24^, we have shown that muography is mature to go beyond the stage of a technique reserved to highly specialized groups and can legitimately aspire to become a standard investigation tool using low-cost instrumentation. At the time, we provided first indications of hidden underground cavities. However, the complexity of the system of cavities underground Mt. Echia makes it difficult to identify hidden cavities without the ambiguities originating from the fact that standard 2D muographies are projective transmission maps, in which the shadows of other cavities are difficult to disentangle from one another. To continue the investigation, we pursued a new development: a novel approach to three-dimensional (3D) muography, where the usual 2D muographies taken from at least three different locations are directly combined in a single analysis in order to localise cavities in space and reconstruct their shape.

The new approach is based directly on muographic data, providing a 3D image with high resolution. The method is suitable for the detection of sharp discontinuities in density related to the presence of cavities and can be applied even to complex systems such as at Mt. Echia and other anthropic environments. Moreover, no a-priori information about the location of the cavities and on the density distributions are required. The success in identifying a hidden cavity at Mt. Echia demonstrates the discovery potential enhancement achievable with our approach to 3D muography, especially in case of complex systems of underground structures.

The 3D reconstructions based on inversion methods reported in previous works^3,21,25–28^ are qualitatively and quantitatively different from the 3D muography developed by us. They are based on the subdivision of the object under investigation in voxels (volume pixels), with a subsequent fit of their density to data. The constraints usually applied to the density of nearby voxels make them suitable in cases where the variations in density are rather smooth and a high resolution is not required. They have typically been applied to volcanoes and other geological structures.

With the intent to make this paper readable by a large audience, we refer to our previous publication on Mt. Echia^24^ for technical details about 2D muography that are of interest to specialists.

## Muon trackers

The two muon trackers (called MU-RAY and MIMA) used for the measurements reported in this paper are real-time electronic devices that operate autonomously, with remote control and readout. The basic elements are bars of plastic material doped with a scintillating compound, following a simple and widespread technique for particle detection. The light generated by muons in the plastic scintillator bars is detected by Silicon Photomultipliers (SiPMs). These photosensors, recently developed, are solid-state devices that, as such, do not require any high voltage supply and have a very low power consumption. These features allow us to operate the detectors with relative ease also in remote environments. For a detailed description of the technology we address the reader to ref.^24^.

**Figure 1:**
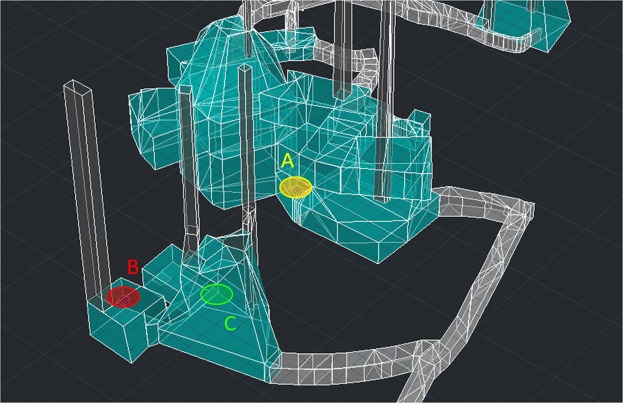
A half module of the MU-RAY muon tracker with its 32 triangular scintillator bars and the 32 wavelength shifting optical fibres that transmit the light to the photosensors.

The basic module of the MU-RAY muon tracker^7,8,24^ is a planar array of 64 plastic scintillator bars. The section of the scintillator bars has the shape of an isosceles triangle with 3.3 cm basis and 1.7 cm height. A central hole hosts a wavelength shifting optical fibre that carries the light to the SiPM. The scintillator bars were extruded at the NICADD facility of the Fermilab Laboratory. The triangular bars are packed together so that each one is half superimposed to its neighbours. The coordinate perpendicular to the bars is measured with a resolution of about 2 mm. The module is subdivided in two arrays of 32 scintillator bars. Figure 1 shows one of such arrays.

**Figure 2:**
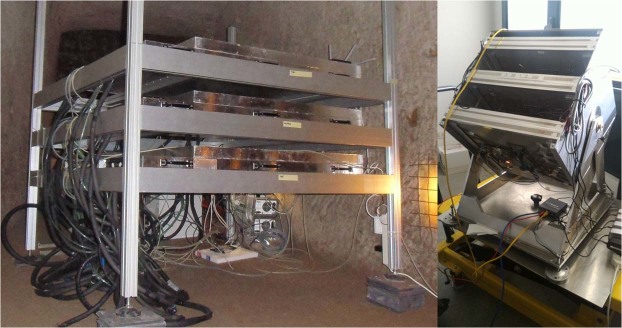
The MU-RAY (left) and MIMA (right) muon trackers.

Two modules with orthogonal bars form a XY plane. The muon trajectories are tracked by a sequence of three XY planes. At Mt. Echia, the distance between the outer planes was 0.50 m. The angular resolution in the measurement of the muon trajectory with this configuration was around 6 mrad. The XY planes have an active area of about 1.0 × 1.0 m. Muons are tracked up to a maximum angle of 60° with respect to the view axis of the tracker, i.e. to the direction perpendicular to the tracker planes. The left side of Fig. 2 shows the MU-RAY tracker at location A.

MIMA^29^ is a smaller muon tracker basically using the same technique, except that the SiPMs are in direct contact with the scintillator bars (i.e. no wavelength shifter is used). Like the MU-RAY tracker, it consists of three XY planes. The outer XY planes are formed by two orthogonal arrays of 21 scintillator bars. The section of the scintillator bars has the shape of an isosceles triangle with 4 cm basis and 2 cm height. The coordinates of the muons are measured with a resolution of about 3 mm. The outer XY planes are 0.34 m apart. Muon tracking is carried out with an angular resolution of about 14 mrad. The central XY plane, with a coarser structure and formed by scintillator bars of rectangular section, is used as a constraint in muon tracking. However, its use was not required for this measurement. Muons are tracked up to a maximum angle of 45° with respect to the view axis of the tracker. The active area amounts to about 0.4 × 0.4 m (1.0 × 1.0 m for MU-RAY). The right side of Fig. 2 shows the MIMA tracker.

## Muon Transmission

Each muon tracker determines the muon trajectories after the traversal of the volume under investigation. The procedure for the analysis of the events with a muon trigger acquired in the data taking runs, in order to select those with a recognizable muon and to reconstruct its track, is described in ref.^24^ for the MU-RAY tracker and in ref.^29^ for the MIMA tracker.

The muon flux is measured as a function of the elevation angle *α* and of the azimuth angle *ϕ* of the track, as defined in the reference system of the tracker itself. The elevation angle (complementary to the zenith angle) is zero in the horizontal direction and 90° at the Zenith.

The muon transmission *T*(*α*, *ϕ*) is defined as the ratio between the muon flux measured underground as described above and the flux measured at the surface in dedicated runs carried out in the laboratory (free sky)^24^. Since no damage to the detectors or change of operating conditions happened during the runs, we assume that the geometrical acceptance, the trigger efficiency, and the selection efficiency are the same in the data samples taken underground and at the surface, effectively cancelling out in the ratio giving the muon transmission. For a detailed mathematical formulation, see the Section Methods or ref.^24^.

The relative transmission *R*(*α*, *ϕ*, *ρ*) is defined as the ratio between the measured and the expected muon transmission, the latter evaluated by taking into account the absorption of the muon flux in the absence of internal structure such as cavities. The relative transmission equals unity in case no internal structures are present and the correct rock density *ρ* is inserted in the evaluation of the expected transmission.

The expected transmission was evaluated using the muon flux as a function of energy measured at the surface by the ADAMO experiment^30^. The muon flux reaching the tracker was evaluated by integrating the flux impinging on the surface above a muon energy threshold that takes into account the energy loss in the rock traversed^31^. The path length of muons in the rock was obtained using a Digital Terrain Model (DTM) describing Mt. Echia. The DTM was obtained from LIDAR observations elaborated with the software packages Terrasolid - Terrascan, LP360, RIEGL Riscan Pro, Golden Software - Surfer 12 and Root^24^. We assumed an uniform density of the rock (tuff at Mt. Echia) and used its best estimate *ρ* = 1.71 g/cm^3^, as previously determined from our data^24^.

## Localization in space of a hidden cavity

**Figure 3:**
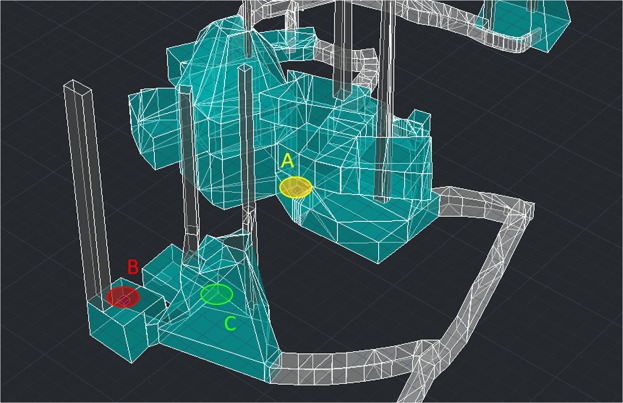
The system of known cavities and the three locations A, B and C of the muon trackers.

**Figure 4:**
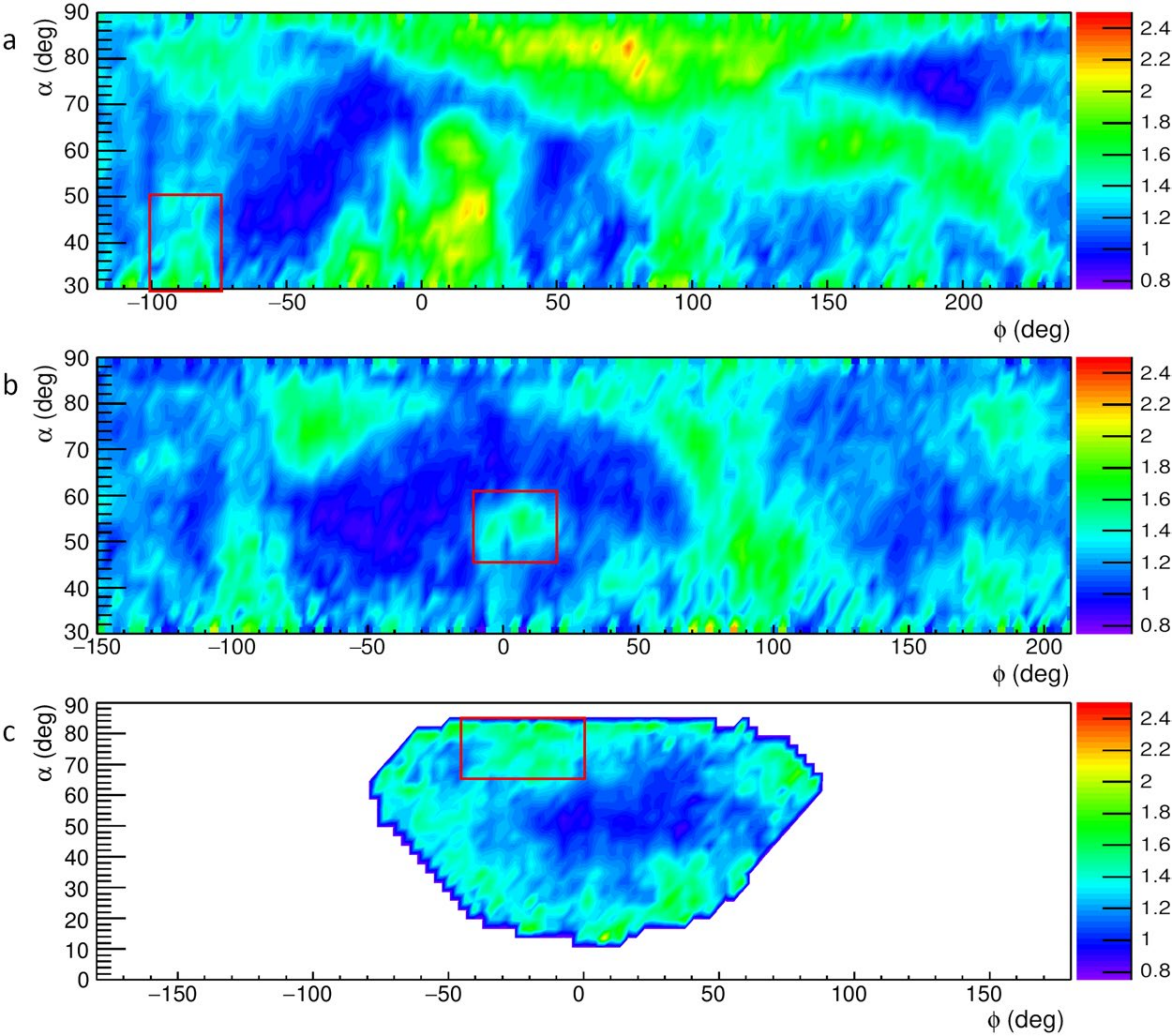
**a**–**c** The relative transmission *R*(*α*, *ϕ*, *ρ*) observed at the locations A, B and C, respectively, in the reference systems of the corresponding muon trackers. The angular regions associated to the hidden cavity are indicated with rectangles. The plot was obtained using the software ROOT and the smoothing tool Contour4 was applied.

Figure 3 shows the system of known cavities inside Mt. Echia visible from the three muon tracker locations A, B and C (indicated in the figure and at an altitude around 10 m a.s.l.). Figure 4a–c shows the relative transmissions *R*(*α*, *ϕ*, *ρ*), as measured at the three locations A, B and C of the muon trackers, each one giving a projective muographic image. Regions with high relative transmission (green in the figure) signal the possible presence of cavities.

The results presented in ref.^24^ were obtained with the MU-RAY muon tracker installed at location A. A second set of data was taken immediately afterwards with the same MU-RAY tracker at the location denoted by B. At both locations, the XY planes of the tracker were placed horizontally parallel to the cavern floor, so that the view axis of the tracker was vertically oriented.

Figure 4a concerns location A and is reproduced from ref.^24^. We focused attention on the (green) signal of high transmission visible near the bottom-left corner, which suggests the presence of a hidden cavity. The high transmissivity region of interest is within an angular boundary indicated by a red rectangle that corresponds to an elevation angle *α*_*A*_ in the range of 30° to 50°, and an azimuth angle *ϕ*_*A*_ in the range of −100° to −75°.

The projection in space of this angular region from location A intercepts the projection from location B of a similar region of high transmissivity that lies within the angular boundary indicated by the rectangle in Fig. 4b. The region corresponds to *α*_*B*_ in the range of 45° to 60° and *ϕ*_*B*_ in the range of −10° to 20°. The muography carried out from location B thus confirmed the presence of a hidden cavity and roughly localized it in space at the intercept of the two projections.

We then took data, using the MIMA tracker, at the location denoted by C in Fig. 3 The view axis of the MIMA tracker was tilted by about 45.5° with respect to the vertical direction and was pointing towards the presumed location of the hidden cavity.

The hidden cavity under investigation is viewed from location C at an angle that is different than that at locations A and B. This choice of the viewing site C effectively allows us to perform a high resolution 3D study in combination with the muographies taken at locations A and B. However, the choice of tracker locations was done compatibly with the logistics of the underground site, where we used pre-existing caves and tunnels. Hence the chosen locations are adequate but not optimal for a 3D muography.

In Fig. 4c a signal for a hidden cavity from location C is seen in the angular region expected from the two previous muographies, This high transmissivity region is contained within the red rectangle, which corresponds to the ranges of 65° to 85° and of −45° to 0° for *α*_*C*_ and *ϕ*_*C*_, respectively. Actually, the three angular regions indicated by rectangles in Fig. 4a–c all point to the same high transmissivity structure (i.e. a hidden cavity).

The MU-RAY tracker data taking at locations A and B lasted 26 and 8 days, respectively. The numbers of acquired events with a muon trigger were 14 × 10^6^ at location A and 5 × 10^6^ at location B. The MIMA tracker data taking at location C lasted 50 days, corresponding to 5 × 10^6^ muon trigger events. The runs to measure the muon flux on the surface were taken keeping the same orientation of the muon trackers as underground. The run lasted 2 days, corresponding to 12 × 10^6^ acquired muon trigger events for MU-RAY, and 18 days, corresponding to 19 × 10^6^ muon trigger events, for MIMA.

## 3D reconstruction of the hidden cavity

A clustering algorithm was applied to select regions in Fig. 4a–c with a relative transmission R above a significant threshold value, so that they could be considered as corresponding to cavities. The clustering algorithm was based on the assumption that the distribution of the relative transmission *R* in Fig. 4a–c has two components. One of the components corresponds to the transmission mostly through solid rock. A second component correspond to cases where voids are encountered by muons. These two components were obtained by fits with Gaussian curves. The component corresponding to transmission through solid rock (essentially determined by the shoulder at low transmission) was used to set the threshold to define the clusters corresponding to the presence of voids. This threshold was set at a value such that the probability to be a solid rock region is smaller than 2.5%. This corresponds to a value of *R* above 1.51 in the muographies acquired with the MU-RAY tracker (Fig. 4a,b) and to a slightly different value (1.52) for the muography with the MIMA tracker (Fig. 4c).

**Figure 5:**
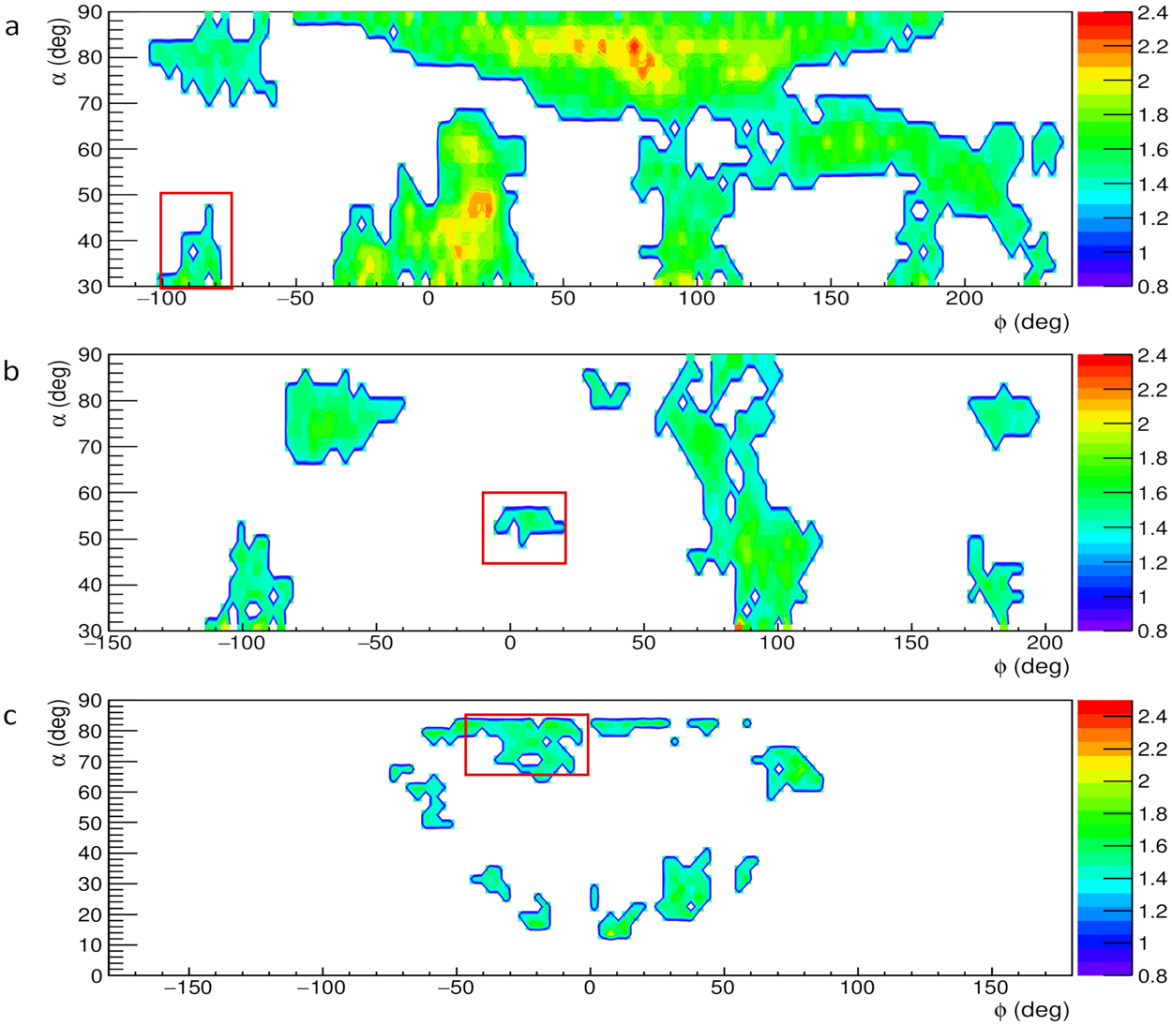
Regions in the map of the relative transmission *R*(*α*, *ϕ*, *ρ*) selected by the clustering algorithm as corresponding to a cavity. The angular regions associated to the hidden cavity are indicated with rectangles. The plot was obtained using the software ROOT and the smoothing tool Contour2 was applied.

The threshold obtained in this manner is used to search for the “seeds” (i.e. the starting regions) of the clustering algorithm. Subsequent recursive aggregation of points appearing in the 2D muography to each cluster is made by lowering progressively the threshold in *R* for points which are close in space to existing clusters. Cluster reconstruction stops when no further points (close in space to an existing cluster) have a high-enough value of *R* (1.37 for MU-RAY and 1.39 for MIMA) to be associated to the cluster. In this second selection the probability for a rock filled bin to exceed the thresholds was higher (16%) but the request of proximity to a signal region ensures a low background. The regions selected by the clustering algorithm are indicated by solid lines in Fig. 5a–c.

In order to reconstruct in space the hidden cavity, we started by defining a grid of points in a cubic volume that encloses the region of space where the cavity is supposed to be, according to the angular ranges defined above. As a general criterion, a point was considered to be located inside a cavity if in each of the three projective muographies it corresponded to a direction lying inside a signal cluster as defined above.

This procedure was tested by simulating the presence of a spherical cavity with 6 m diameter, approximately located at the presumed position of the hidden cavity and having a similar size. The simulated cavity was surrounded by rock using our best estimate of density for Mt. Echia^24^ as quoted above in this paper. The minimum cavity size for our experiment sensitivity, was estimated to be 1.5 m over 50 m total rock thickness^24^. The spherical shape was chosen in order to study the effects of the non optimal choice of the trackers’ locations, and in particular to highlight possible ensuing asymmetries.

**Figure 6:**
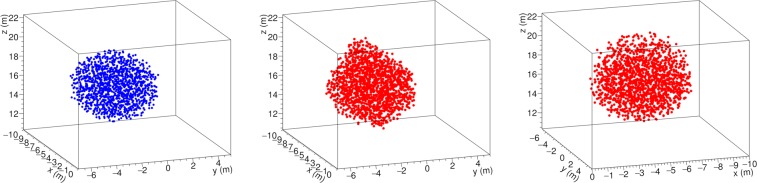
The simulated spherical cavity with a 6 m diameter (blue dots, on the left) and two views of its 3D reconstruction (red dots, on the right), in a coordinate system with origin at the centre of the MIMA muon tracker at location C.

The result of the simulation is shown in Fig. 6. The blue dots in the view on the left side represent an ensemble of points distributed on the grid and located inside the simulated spherical cavity. The points satisfying the triple cluster criterion are denoted by red dots in the two views on the right side of the figure.

The criterion is satisfied also by points that in a projection appear in the shadow of the cavity, rather than inside. The halo that is observed in Fig. 6, extends by about 1 m beyond the cavity. In particular, one can notice a halo upwards, presumably due to fact that the three tracker locations are all at a level considerably lower than the hidden cavity (hence of the spherical cavity). The halo could be reduced by placing the trackers at angles that are more favourable for a triangulation or by increasing the number of trackers locations beyond the minimal number of three used in this work.

**Figure 7:**
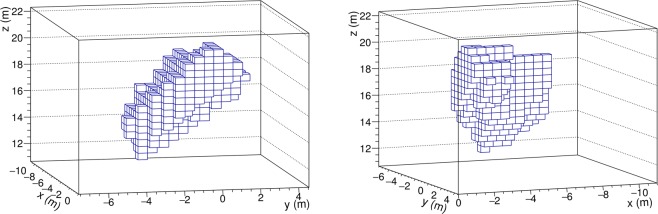
Two views of the 3D reconstruction of the hidden cavity, in a coordinate system with origin at the centre of the MIMA muon tracker at location C.

**Figure 8:**
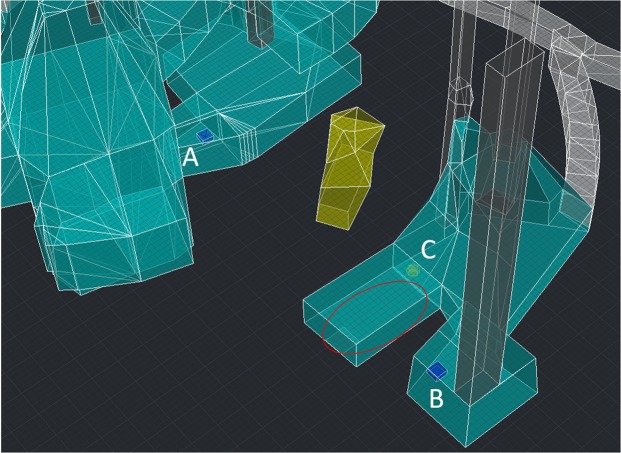
The 3D reconstruction of the hidden cavity (in yellow) inserted in the CAD model. The ellipse indicates the cavity where debris were found, providing a hint for a hidden cavity above it.

Figure 7 shows two views of the 3D muographic image of the hidden cavity in the reference system associated to the MIMA muon tracker, as obtained following the procedure described above. It appears as an inclined cavity with a width of about 4 m, a height of 3–4 m and a length of about 7 m. Figure 8 shows the hidden cavity inserted in the CAD representation of the known cavities.

Especially when compared with the single muographies given in Fig. 4a–c, taken as examples of current 2D muographic images, the reconstruction in space of the hidden cavity shown in Fig. 7 demonstrates the power of our approach to 3D muography in identifying, localizing and reconstructing in space hidden cavities in complex systems, resolving the the ambiguities that affect 2D muographies.

## Hints for the hidden cavity

The existence of a hidden cavity in the region of space localised by our 3D muography, is consistent with previous hints from surveys carried out on site by one of the present authors (G. Minin), together with personnel of the Bourbon Tunnel. The cavity outlined with the ellipse in Fig. 8 was found to be obstructed by a large quantity of material that could not be removed. The presence of this material suggested the existence of an empty space at a higher altitude, originally belonging to a larger sized structure. Various unsuccessful attempts have been made at finding accesses to the cavity from the top or from the sides. Also unsuccessful were the attempts made at probing the hidden cavity from neighbouring cavities with a ground-penetrating radar (GPR), having a reach limited to about 3 m^32^.

Presumably, the hidden cavity was originally a quarry from where tuff was extracted to construct a building above it. Subsequently it was probably turned into a cistern, and tied up to branches of the aqueduct dating from the Renaissance. Clues have been found, indicating that during the Second World War work had started to access the underground shelters from the surface through a staircase, presumably within this hidden cavity. After the war, the cavity was partially filled with debris, on several occasions.

## Outlook

The hidden cavity was reconstructed in space on the basis of its correspondence with the presence of a high muon transmittivity cluster in each of the three projective images. These clusters were defined through the use of algorithms based on setting a threshold in the transmittivity. The technique could be further refined through the development of algorithms that fully exploit the quantitative information carried by the transmittivity.

We also developed and started to apply an innovative technique that allows a first localisation in space already from a single muography^33^. This technique is based on a stereoscopic principle similar to that of the human sight and is applicable wherever the size of the muon tracker is not negligible with respect to its distance to the structure being investigated. In this case, a rough estimate of this distance can be obtained by projecting backwards the muographic image of the object and minimizing its angular width as a function of the distance of the plane onto which it is projected.

Our group is now planning the study of underground structures in the mountain where the ancient city of *Cumae* was settled as a Greek colony in the 8th century BC and in the surrounding area of the Phlegraean Fields, very rich of archaelogical remains. The Phlegraean Fields were of primary strategic importance in the Roman times, with the commercial harbour at *Puteoli* and the military harbour at *Misenum*. Moreover, *Baia* was hosting a rich residential and thermal area. In addition, a measurement campaign with MIMA tracker is ongoing inside the Temperino archaeological park, a mine from the Etruscan age located in Campiglia Marittima (Tuscany).

## Conclusions

We have developed a new method for a 3D muographic reconstruction directly from the data, exploiting the resolution obtainable with the muographic technique. We have proven the existence of a hitherto unknown cavity inside Mt. Echia, of which only hints were present, and reconstructed its position and shape in space. The search at Mt. Echia was motivated both by the need to investigate an area of potential geological risk and by the curiosity about the history hidden underneath the ancient city of Naples.

Because of its complex system of cavities, Mt. Echia also represents a testing ground fraught with difficulties in which to develop muography techniques for application in the field of archaeology and, in general, in the investigation of hidden structures. Especially in complex situations, our 3D muographic reconstruction method definitely enhances the discovery potential of the technique.

## Methods

### Muon transmission

For any given angular bin (*α*, *ϕ*), the muon transmission T is defined as the ratio between the muon rate observed underground and the rate observed on the Earth surface. The latter is measured in a dedicated run at a surface laboratory without overburdens or overhead structures (free sky run)^24^.

As the muon tracker and its operating conditions are the same at the two locations, most factors (such as the sensitive area of the muon tracker, its angular acceptance, the trigger efficiency and the analysis efficiency) linking the muon rates to the number of recorded events *N*(*α*, *ϕ*) cancel out in the ratio. This is not the case for the data taking time Δ*T* and for the data acquisition efficiency *ϵ*_*DAQ*_(*ν*), which depends of the trigger rate *ν* that is much lower underground.

With good approximation the measured muon transmission *T*^*m*^(*α*, *ϕ*) is thus given by

1 $${T}^{m}(\alpha ,\varphi )=\frac{{\rm{\Delta }}{T}^{fs}}{{\rm{\Delta }}{T}^{u}}\cdot \frac{{{\epsilon }}_{DAQ}({\nu }_{fs})}{{{\epsilon }}_{DAQ}({\nu }_{u})}\cdot \frac{{N}^{u}(\alpha ,\varphi )}{{N}^{fs}(\alpha ,\varphi )}$$

where the superscripts or subscripts *u* and *fs* indicate quantities referring to the underground and to the free sky data taking, respectively.

The muon transmission *T*(*α*, *ϕ*, *ρ*) that is expected in the absence of internal structures (such as cavities) is given by

2 $$T(\alpha ,\varphi ,\rho )=\frac{{\int }_{{E}_{\min }}^{\infty }\,{\rm{\Phi }}(\alpha ,E)dE}{{\int }_{{E}_{0}}^{\infty }\,{\rm{\Phi }}(\alpha ,E)dE}$$

where Φ(*α*, *E*) is the differential muon flux impinging on the Earth surface as a function of the muon energy *E*, which also depends on the elevation angle *α* and has been measured by the ADAMO experiment^30^. The lower limit *E*_*min*_(*α*, *ϕ*, *ρ*) of the integral at the numerator is the minimum energy that muons must have in order to cross the rock overburden and be recorded underground by the muon tracker. It depends on average rock density *ρ* and on the rock thickness, the latter being given by the DTM for any given (*α*, *ϕ*). *E*_*min*_ is evaluated using a model that parametrises the muon energy loss in matter^31^. The lower limit *E*_0_ of the integral at the denominator is the minimum energy required for muons to be recorded by the muon tracker. For the MU-RAY muon tracker it is estimated at about 100 MeV, supposedly independent on (*α*, *ϕ*) in the angular range under study. The sensitivity of the expected muon transmission on *E*_0_ is such that a value of 200 MeV would result in a transmission higher by at most 4%. The estimate for the MIMA muon tracker is *E*_0_ = 170 MeV.

The relative muon transmission *R*(*α*, *ϕ*, *ρ*) is defined as

$$R(\alpha ,\varphi ,\rho )=\frac{{T}^{m}(\alpha ,\varphi )}{T(\alpha ,\varphi ,\rho )}$$

and represents for each angular bin, the muon excess (or deficit) for the target under observation when compared to a uniformly homogeneous rock hypothesis.

### Clustering algorythm

**Figure 9:**
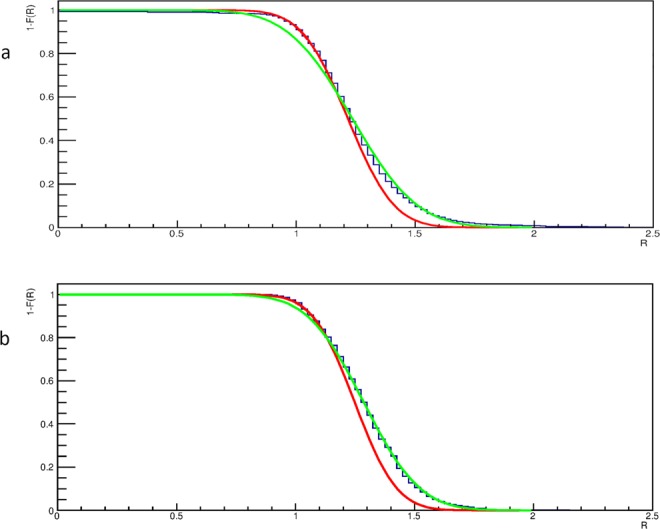
Fits of the cumulative distributions of the relative transmission *R* of the muography taken with the MU-RAY muon tracker at the location B (**a**) and with the MIMA muon tracker (**b**). The distributions are fitted by two Gaussian components, one corresponding to transmission through rock without voids (red) and another corresponding to trasmission through rock with voids (green).

The distribution of the relative transmission *R* given in each muography of Fig. 4a–c was interpreted as the sum of two components, one corresponding to transmission through rock without voids and another corresponding to transmission through rock with voids. We complement the main text of the paper showing in Fig. 9a,b fits with two Gaussian components. The fits are shown for one of the locations of the MU-RAY muon tracker and for the MIMA muon tracker.
