# Author Correction: 3D Muography for the Search of Hidden Cavities

**DOI:** 10.1038/s41598-019-43833-z

**Published:** 2019-05-09

**Authors:** Luigi Cimmino, Guglielmo Baccani, Pasquale Noli, Lucio Amato, Fabio Ambrosino, Lorenzo Bonechi, Massimo Bongi, Vitaliano Ciulli, Raffaello D’Alessandro, Mariaelena D’Errico, Sandro Gonzi, Barbara Melon, Gianluca Minin, Giulio Saracino, Luca Scognamiglio, Paolo Strolin, Lorenzo Viliani

**Affiliations:** 1 University of Naples Federico II., Naples, Italy; 2 INFN sezione di Napoli., Naples, Italy; 3 University of Florence, Florence, Italy; 4 INFN sezione di Firenze, Florence, Italy; 5 TECNO-IN S.P.A., Naples, Italy; 6 Associazione Culturale Borbonica Sotterranea, Naples, Italy

Correction to: *Scientific Reports* 10.1038/s41598-019-39682-5, published online 27 February 2019

In this Article, Figure 1 is a duplication of Figure 3. The correct Figure 1 appears below.

**Figure 1:**
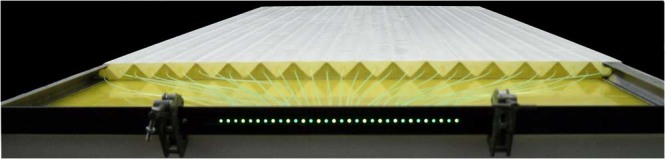
A half module of the MU-RAY muon tracker with its 32 triangular scintillator bars and the 32 wavelength shifting optical fibres that transmit the light to the photosensors.

